# Hypertension-Related Admissions and Outcome in a Tertiary Hospital in Northeast Nigeria

**DOI:** 10.1155/2012/960546

**Published:** 2012-06-19

**Authors:** P. M. Kolo, Y. B. Jibrin, E. O. Sanya, M. Alkali, I. B. Peter Kio, R. K. Moronkola

**Affiliations:** ^1^Department of Medicine, University of Ilorin Teaching Hospital, PMB, Ilorin 1459, Nigeria; ^2^Department of Medicine, Abubakar Tafawa Balewa University Teaching Hospital, PMB, Bauchi 0117, Nigeria

## Abstract

Cardiovascular disease has reached near epidemic proportion in sub-Saharan Africa, and systemic hypertension (SH) remains the driver of cardiovascular complications. We studied hypertension-related admissions and their outcome at the Abubaker Tafawa Balewa University Teaching Hospital (ATBUTH) Bauchi, Northeast Nigeria. Records of all patients admitted into the medical wards between 1st November 2010 and 31st October 2011 were studied, and case files of those managed for SH complications were selected for detailed examination. Of the total 3108 admissions, 735 (23.7%) were hypertension related. Mean systolic blood pressure (SBP) and diastolic blood pressure (DBP) were 167.4 ± 18.2 and 98.6 ± 13.5, respectively, at presentation. Although, hypertension-related admissions were 23.7% of total admissions, there was an excess of mortality associated with SH complications (42.9%). Stroke was the commonest, and it accounted for 44.4% of cases. Stroke had the highest mortality (39.3%), followed by chronic kidney disease (36.6%); hypertensive emergencies (30.9%) and hypertensive heart failure had the lowest intrahospital mortality (27.5%). In conclusion, SH-related admissions are common among medical admissions in Bauchi Nigeria and are associated with high mortality. Community interventions that promote early diagnosis and reduction of cardiovascular risk profiles are urgently needed to reduce SH deaths.

## 1. Introduction

According to the new report from World Health Organization (WHO) published in September 2011, cardiovascular diseases (CVDs) remain the leading cause of death and disability in the world [1]. Noncommunicable diseases accounted for more than 36 million deaths in 2008 with CVDs responsible for 48% of these deaths, cancers 21%, chronic respiratory diseases 12%, and diabetes mellitus 3%. Over 80% of CVD deaths occur in low- and middle-income countries. Although, a large proportion of CVDs is preventable, they continue to rise mainly because preventive measures are inadequate and it has been projected that by year 2030, almost 23.6 million people will die from CVDs [1]. Similarly, majority of CVD deaths in low income countries occur in individuals less than 60 years of age [1, 2]. These premature deaths have grave economic and social implications. Interestingly, while actions to reduce blood pressure and cholesterol are having an impact on overall CVD mortality in high-income countries, there is worsening of CVD risk profiles in most developing regions of the world [3].

In sub-Saharan Africa, the prevalence of CVDs has reached near epidemic proportions with SH being the main driver of cardiovascular complications [4]. Whereas SH was said to be rare in Africans in the first half of the twentieth century, current evidences have shown it affects between 30 and 60% of Black Africans [5]. It is the commonest cause of heart failure, stroke, and kidney disease from many studies in Africa. This upsurge in the incidence of SH and its complications in sub-Saharan Africa with high burden of infectious diseases and poverty had greatly reduced the life expectancy in this part of the world [6]. Although there are indications that SH plays a major role in cardiovascular outcomes, there is paucity of data particularly in the Northeast Nigeria on mortality profile of hypertension-related admissions into the medical wards, hence this study.

## 2. Materials and Methods

This is a retrospective study which reviewed the frequency and outcome of hypertension-related admissions at the ATBUTH, Bauchi North East Nigeria. The hospital is a 650-bedded tertiary health care facility established in 2009 with 130 beds dedicated to medical admissions. The health facility serves as a referral centre for the residents of Bauchi State with estimated population of 4.7 million (2006 Nigerian Population Census) and neighbouring states of Gombe, Yobe, and Adamawa.

Diagnoses and management of all patients admitted into the Emergency Unit are usually discussed the next day at the morning review with all consultants in attendance. This is to ensure accurate diagnosis of cases and to provide quality care to the patients. Blood pressure was measured in the emergency department using a standard mercury sphygmomanometer (Accoson) every 30 minutes until it was stable. Average of first three recordings was taken as blood pressure value of the patient. Hypertension was defined as admission blood pressure of ≥140/90 mmHg or antihypertension medication usage [7].  Records of all patients admitted between 1st November 2010 and 31st October 2011 were studied. Although we independently reassessed patients' diagnoses from their medical records, the final diagnosis used in this study was that of the managing consultants. This was because there was high correlation between reviewed diagnoses and the initial one by the managing team. Case files of those admitted for various complications of SH were retrieved using both nurses and medical records admission books. Necessary information was extracted from the case files and entered into a proforma designed for the study. Patients who were admitted for hypertensive heart failure, stroke, or transient ischemic attack (TIA) due to SH, hypertensive nephrosclerosis and hypertensive emergencies or urgencies were included. Excluded from the study were cases with blood pressure less than 140/90 mmHg at presentation and who were not on antihypertensive medication and no documented stigmata of long standing SH. Information obtained from the case notes was patient's age and gender, complications of SH, awareness of hypertension, duration of hypertension, drug compliance, alcohol use, and cigarettes smoking. Others included patient's weight, height, body mass index, SBP, DBP, duration on admission and outcome. Data obtained were analyzed using the SPSS Version 15. Total number of admissions and hypertension-related admissions were noted, and the percentage of latter was calculated. A simple frequency distribution of SH complications was generated. Numerical values were presented as mean ± standard deviation. Student **t*-*test was used to compare means of continuous variables while chi-square test was used to compare means of proportions. Test of correlation was done using the appropriate correlation method. Logistic regression analysis was used to determine predictors of outcome. A statistically significant association was taken at *P* < 0.05.

## 3. Results

Three thousand one hundred and eight patients consisting of 1603 (51.6%) males and 1505 (48.4%) females were admitted at the medical wards of ATBUTH during the period of the study. A total of 588 deaths were recorded giving percentage mortality of 18.9%. Three hundred and fifty-two deaths occurred in males and 236 in females with percentage mortality being higher (*P* = 0.0001) in males (21%) than in females (15.7%).

Of the total 3108 admissions, 735 (23.7%) were due to hypertension-related complications, with mean age of 51.9 ± 17.5 years. Other cardiovascular admissions are displayed in Table 1. Diabetes complications with SH as comorbidity were seen in 96 (3.1%) patients, peripartal cardiomyopathy in 51 (1.64%), and stroke in the young not related to SH was diagnosed in 25 (0.8%) patients. Others included dilated cardiomyopathy in 18 (0.6%) patients and coronary artery in 2 (0.06%) patients. A total of 1220 (39.3%) patients were admitted for cholera which ravaged Bauchi State during the period of the study. When ****cholera patients were excluded from analysis, hypertension-related cases accounted for 38.9% of remaining admissions.

**Figure 1:**
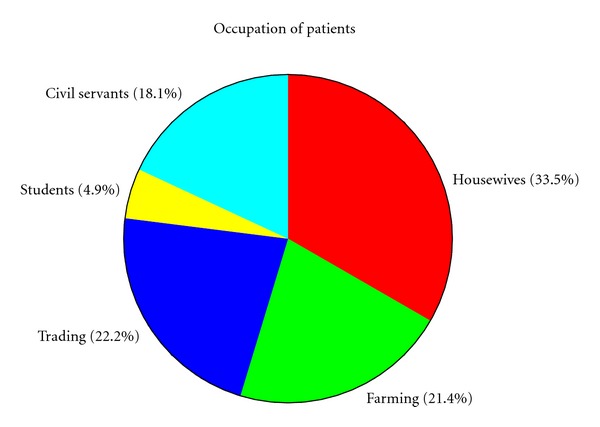
Shows occupational status of the patients.

Occupational status of the patients is presented in Figure 1. Three main occupations among the patients admitted included housewife (33.5%), trading (22.2%), and farming 21.4%). A significant number of the patients were civil servants (18.1%).

Although hypertension-related admissions were 23.7% of total admissions, there was an excess of mortality from SH complications (42.9%). Stroke/TIA was the commonest complication of SH in the patients, and it accounted for 44.4% of cases. This was followed by hypertensive heart failure (27.8%), hypertensive emergencies (16.7%), and chronic kidney disease (11.2%). Mean SBP and DBP were 167.4 ± 18.2 and 98.6 ± 13.5 at presentation. Although 498 (67.8%) patients were aware of their SH status at presentation, only 269 (36.4%) were compliant with their antihypertensive medications.

Comparison of baseline characteristics of survivors and nonsurvivors of SH admissions is presented in Table 2. Nonsurvivors were older (*P* = 0.001) and had longer duration of hypertension (*P* = 0.041) and higher body mass index than survivors. Similarly, pulse rate, SBP, and DBP were significantly higher in nonsurvivors than survivors (*P* = 0.001, 0.001 and 0.001, resp.). The mean duration of hospital stay was significantly shorter in nonsurvivors (6.6 ± 7.8 days) in comparison to survivors (11.7 ± 13.6 days), *P* = 0.001. The percentages for awareness of SH, compliance with antihypertensive medications, alcohol use, and cigarette smoking were similar between survivors and non-survivors. Case fatality of hypertension-related admissions was 34.3%. Stroke had the highest mortality (39.3%), followed by chronic kidney disease (36.6%) hypertensive emergencies (30.9%) and hypertensive heart failure had the lowest intrahospital mortality (27.5%). 

Gender differences in mortality profile are presented in Table 3. While duration of SH and pulse rate at presentation was higher in males than in females, the mean body mass index was higher in females. On the other hand, mean age, duration on admission, SBP, and DBP were similar between the two groups. Although mortality profile due to stroke, hypertensive heart failure, and chronic kidney disease as well as hypertensive emergencies was higher in males than females, the differences did not reach statistical significance. 

Multivariable-adjusted odds ratios for mortality are presented in Table 4. The probability of death increased with age (odds ratio = 1.024, *P* = 0.02), pulse rate at admission (odds ratio = 1.02, *P* = 0.001), and duration on admission (odds ratio = 0.957, *P* = 0.002).

## 4. Discussion

The findings from this study showed that hypertension-related admissions accounted for sizeable proportion (23.7%) of medical admissions among adult patients from a tertiary health institution in Northeastern Nigeria. This result is comparable to 20.2% [8] from another tertiary hospital in Southsouth and 18.4% from Southeastern Nigeria [9]. Similarly Ndjeka et al. reported 19% [10] from a rural hospital in South Africa. Interestingly, when cases of acute cholera admissions were excluded from the data because cholera was epidemic during the study period, the proportion of hypertension-related admissions rose to 38.9%. The three commonest complications of SH were stroke (44.4%), hypertensive heart failure (27.8%), and hypertensive emergencies (16.7%), respectively. Although hypertension-related complications accounted for 23.7% of medical admissions, mortality related to this condition was 42.9%. This mortality profile is high and may be a reflection of severity of SH complications in the patients. 

Casefatality of hypertensive-related admissions was 34.3%, and this was significantly influenced by age, duration of SH, body mass index, and admission pulse rate. Others were SBP and DBP as well as duration on admission. Factors that were predictive of mortality in this study were patients' age, admission pulse rate, and duration of admission. Some of these factors have also been identified in a study among Congolese [11]. 

The overall mean length of hospital stay was 10.1 ± 12.7 days (median 7 days). Non-survivors had a median duration of 4 days at death compared to survivors with median stay of 8 days, and most deaths occurred within the first week of admission. Mean duration of hospital stay has been documented in some studies to be associated with mortality [12]. One important finding of this study is the fact that two-thirds of the patients had been diagnosed as being SH prior to admission. Among those who were aware of their SH status, drug compliance rate was 54%. This proportion is similar to what had been reported in other studies in Africa [11, 13] and may be one of the reasons for high mortality in the region. High level of illiteracy along with poverty and side effects of medications was among several reasons given for poor compliance. Unlike the study from Congo where death was associated with younger age, report of this study showed that hypertension-related death was more in older people. The tendency of older people having more cardiovascular comorbid conditions could partly explain this observation. High pulse rate was another factor that predicted risk of death from complication of SH in this study. Several published data have demonstrated a positive association between pulse rate and all-cause mortality in hypertensive individuals [14, 15]. This has an important implication for choice of medication especially in the presence of complications. Although smoking and alcohol abuse had been associated with poor outcomes in hypertensive patients, findings from this study did not show any significant difference amongst survivors and non-survivor in this regard. 

In Europe and America, hypertensive cardiovascular complications are commonly seen in older age group [16, 17]; however, relatively younger individuals were affected in our study. This has negative economic implications for the individuals affected and their families. Majority of our patients were not covered by the National Health Insurance scheme which has just started in Nigeria and thus had to pay for drugs and services at the point of care which increase out-of-pocket expenses. In addition, a sizeable number of our patients were housewives and students with little or no sources of income. These reduce their financial capability to purchase antihypertensive medications and compliance with their treatment. 

Stroke was the commonest complication of SH amongst this group of Nigerians, and this agrees with previous studies from other regions in Nigeria [8, 9, 17]. Recent community and hospital-based data have shown increasing cases of stroke in Africa [18, 19]. This has been attributed mainly to high prevalence of uncontrolled SH [18]. On the other hand reports from high income countries seem to suggest a decline in hypertension-related complications [20]. Part of the reasons for this is availability of potent antihypertensive medications with minimal side effects which are associated with greater compliance. 

Gender was another factor that influenced outcome of patients in this series. Mortality among males (21%) was significantly higher than in females (15.7%). Gender-related difference in mortality among medical admissions had been reported from a hospital-based study in Nigeria [21]. Although females tend to use hospital more frequently, male patients often present with complications. 

Part of the limitations of this study includes the fact that it was a retrospective study and being hospital based. It might be difficult to generalize our result to the entire community since study was from a tertiary hospital which serves as a referral centre to the neighbouring states. Low postmortem rate to confirm or refute diagnoses was another limitation of the study. 

In conclusion, SH complications are common among medical admissions in Bauchi Nigeria and occur at a relatively young age. Hypertension-related admissions are associated with excess mortality, and stroke is the commonest. Community intervention to health-educate the populace on the need for early detection, dangers of SH, and other cardiovascular risk factors is urgently needed. Women empowerment programs and provision of gainful employment by the government will reduce poverty and improve medication compliance among hypertensive individuals.

## Figures and Tables

**Table 1:** Shows spectrum of cardiovascular admissions in the medical wards of ATBUTH.

Causes	Number	Percentage (%)
Hypertension-related	735	23.6
Diabetic complications	96	3.1
Peripartal heart failure	51	1.64
Stroke in the young	25	0.8
Dilated cardiomyopathy	18	0.6
Cor pulmonale	9	0.3
Rheumatic heart disease	3	0.1
Congenital heart disease	2	0.06
Myocardial infarction	2	0.06
Cholera	1220	39.3
Others	947	30.1
Total	3108	100

**Table 2:** Shows comparison of baseline characteristics of survivors and nonsurvivors of hypertension admissions.

Parameter	survivors (Mean ± SD)	non-survivors (Mean ± SD)	*P*-value
Number (%)	483 (65.7)	252 (34.3)	
Age (years)	53.9 (15)	58.4 (15.4)	0.001^∗^
Duration of SH (years)	7.6 (7.5)	8.8 (8.1)	0.041^∗^
Weight (kg)	65.9 (8.8)	68.6 (11.4)	0.002^∗^
Height (m)	1.68 (0.07)	1.69 (0.07)	0.28
BMI (kg/m^2^)	23.3 (2.5)	24.1 (3.5)	0.01^∗^
Pulse (beats/min)	82.4 (15.4)	87.6 (17.9)	0.001^∗^
SBP (mmHg)	161.6 (15.4)	169.5 (17.9)	0.001^∗^
DBP (mmHg)	97.5 (9.9)	101.8 (13.5)	0.001^∗^
DOA (days)	11.7 (13.6)	6.6 (7.8)	0.001^∗^
HBP awareness (%)	326 (67.5)	172 (68.3)	0.8
Drug compliance (%)	179 (37.1)	90 (35.7)	0.7
Alcohol use (number %)	57 (11.8)	32 (12.7)	0.7
Smoking (number %)	16 (3.3)	9 (3.6)	0.85
Diabetes (number %)	89 (18.4)	53 (21.0)	0.4
Stroke/TIA (number %)	198 (60.7)	128 (39.3)	
HHF (number %)	148 (72.5)	56 (27.5)	
HBP emergencies (%)	85 (69.1)	38 (30.9)	
CKD (number %)	52 (63.4)	30 (36.6)	

SD: standard deviation, SH: systemic hypertension, BMI: body mass index, SBP: systolic blood pressure, DBP: iastolic blood pressure, DOA: duration on admission, TIA: transient ischaemic attack, HHF: hypertensive heart failure, CKD: chronic kidney disease.

^∗^Statistically significant.

**Table 3:** Gender differences in mortality of hypertension-related admissions.

Parameter	Males	Females	*P*-value
	Mean ± SD	Mean ± SD	
Age (years)	52.9 ± 17	51.1 ± 17.9	0.12
Duration of SH (years)	9.0 (8.7)	6.8 (6.4)	0.001^∗^
BMI (kg/m^2^)	23.3 (2.4)	23.7 (3.2)	0.044^∗^
DOS (days)	9.7 ± 11.3	10.4 ±13	0.39
Pulse (beats/min)	86 (18.9)	82.4 (15.0)	0.004^∗^
SBP (mmHg)	167.9 ± 18.3	166 ± 18.6	0.41
DBP (mmHg)	98.9 ± 13	97.8 ± 12.4	0.5
Stroke (number, % mortality)	187 (40.1%)	139 (38.1%)	0.72
HHF (number, % mortality)	79 (31.6%)	125 (24.8%)	0.29
CKD (number, % mortality)	50 (40%)	32 (31.3%)	0.42
HBPEM (number, % mortality)	59 (33.9%)	64 (28.1%)	0.49
Total (number, % mortality)	375 (37.3%)	360 (31.1%)	0.07

SH: systemic hypertension, BMI: body mass index, SBP: systolic blood pressure, DBP: diastolic blood pressure, DOS: duration of stay in the hospital, HHF: hypertensive heart failure, CKD: chronic kidney disease, HBPEM: hypertensive emergencies.

**Table 4:** Multivariable-adjusted odds ratios for total mortality.

Parameter	Odds ratio (95% CI)	*P*-value
Age (years)	1.024 (1.004–1.044)	0.02
Pulse rate (beats/minute)	1.02 (1.009–1.033)	0.001
DOA (days)	0.957 (0.931–0.984)	0.002

CI: confidence interval, DOA: duration on admission after hospitalization. The variables not entering the logistic regression model were duration of SH, sex, weight, body mass index, SBP, DBP, aware versus unaware of SH, alcohol drinking, and smoking.

## References

[1] World Health Organization (WHO) Non-communicable diseases country profiles 2011. http://whqlibdoc.who.int/publications/2011/9789241502283_eng.pdf.

[2] Opie LH, Seedat YK (2005). Hypertension in sub-Saharan African populations. *Circulation*.

[3] Onwubere BJC, Ejim EC, Okafor CI (2011). Pattern of blood pressure indices among the residents of a rural community in South East Nigeria. *International Journal of Hypertension*.

[4] Kadiri S (2005). Tackling cardiovascular disease in Africa. *British Medical Journal*.

[5] Odili AN, Richart T, Thijs L (2011). Rationale and design of the newer versus older antihypertensive agents in African hypertensive patients (NOAAH) trial. *Blood Pressure*.

[6] Murray CJL, Lopez AD (1997). Mortality by cause for eight regions of the world: global burden of disease study. *The Lancet*.

[7] Kearney PM, Whelton M, Reynolds K, Muntner P, Whelton PK, He J (2005). Global burden of hypertension: analysis of worldwide data. *The Lancet*.

[8] Ukoh VA (2007). Admission of hypertensive patients at the university of Benin teaching hospital, Nigeria. *East African Medical Journal*.

[9] Ike SO (2009). Prevalence of hypertension and its complications among medical admissions at the University of Nigeria Teaching Hospital, Enugu (Study 2). *Nigerian Journal of Medicine*.

[10] Ndjeka NO, Ogunbanjo GA (2003). Disease patterns in the medical wards of a rural South Africal hospital. *South African Family Practice*.

[11] Donnison C (1929). Blood pressure in the African natives: its bearing upon aetiology of hyperpiesa and arteriosclerosis. *The Lancet*.

[12] Garko SB, Ekweani CN, Anyiam CA (2003). Duration of hospital stay and mortality in the medical wards of Ahmadu Bello University Teaching Hospital, Kaduna. *Annals of African Medicine*.

[13] Fuentes R, Ilmaniemi N, Laurikainen E, Tuomilehto J, Nissinen A (2000). Hypertension in developing economies: a review of population-based studies carried out from 1980 to 1998. *Journal of Hypertension*.

[14] Palatini P, Benetos A, Julius S (2006). Impact of increased heart rate on clinical outcomes in hypertension: implications for antihypertensive drug therapy. *Drugs*.

[15] Palatini P (2006). Heart rate: a cardiovascular risk factor that can no longer be ignored. *Giornale Italiano di Cardiologia*.

[16] Van Pelt S, Jauch EC Average age at first stroke decreases in United States but not Italy. http://www.medscape.com/viewarticle/730149.

[17] Ogun SO, Adelowo OO, Familoni OB, Jaiyesimi AE, Fakoya EA (2000). Pattern and outcome of medical admissions at the Ogun State University Teaching Hospital, Sagamu: a three year review. *West African Journal of Medicine*.

[18] Mayosi BM, Flisher AJ, Lalloo UG, Sitas F, Tollman SM, Bradshaw D (2009). The burden of non-communicable diseases in South Africa. *The Lancet*.

[19] Sanya EO, Abiodun AA, Kolo P, Olanrewaju TO, Adekeye K (2011). Profile and causes of mortality among elderly patients seen in a tertiary care hospital in Nigeria. *Annals of African Medicine*.

[20] Whelton PK (1982). Declining mortality from hypertension and stroke. *Southern Medical Journal*.

[21] Kolo PM, Chijioke A (2009). Gender disparities in mortality among medical admissions of a tertiary health facility in Ilorin, Nigeria. *The Internet Journal of Tropical Medicine*.

